# Instant availability of patient records, but diminished availability of patient information: A multi-method study of GP's use of electronic patient records

**DOI:** 10.1186/1472-6947-8-12

**Published:** 2008-03-28

**Authors:** Tom Christensen, Anders Grimsmo

**Affiliations:** 1 Norwegian EHR Research Centre, Faculty of Medicine, Norwegian University of Science and Technology in Trondheim, MTFS, 7489 Trondheim, Norway

## Abstract

**Background:**

In spite of succesful adoption of electronic patient records (EPR) by Norwegian GPs, what constitutes the actual benefits and effects of the use of EPRs in the perspective of the GPs and patients has not been fully characterized. We wanted to study primary care physicians' use of electronic patient record (EPR) systems in terms of use of different EPR functions and the time spent on using the records, as well as the potential effects of EPR systems on the clinician-patient relationship.

**Methods:**

A combined qualitative and quantitative study that uses data collected from focus groups, observations of primary care encounters and a questionnaire survey of a random sample of general practitioners to describe their use of EPR in primary care.

**Results:**

The overall availability of individual patient records had improved, but the availability of the information within each EPR was not satisfactory. GPs' use of EPRs were efficient and comprehensive, but have resulted in transfer of administrative work from secretaries to physicians. We found no indications of disturbance of the clinician-patient relationship by use of computers in this study.

**Conclusion:**

Although GPs are generally satisfied with their EPRs systems, there are still unmet needs and functionality to be covered. It is urgent to find methods that can make a better representation of information in large patient records as well as prevent EPRs from contributing to increased administrative workload of physicians.

## Background

Norwegian GPs started to move their clinical documentation work from paper to EPR systems in the beginning of the 1980's. In the last decade more than 95% of Norwegian GPs have been using an EPR system (personal communication). The high uptake of EPR systems may be looked upon as a proof of their value, but what constitutes the actual benefits and effects of the use of EPRs for GPs and patients have not been fully characterized. Evaluation of EPRs can provide developers, clinicians, and administrators with important information about success and failure [1]. Simultaneous access for multiple users and improved readability compared to handwriting are obvious advantages. Other benefits are flexible visualization of patient data, automated collection of data from accessory medical equipment, automated search, and the generation of reports in different formats. Potential disadvantages can be numerous, such as cumbersome data entry, insufficient overview over the patient's data, nonintuitive interface layouts and erroneous software or hardware [2].

Efficient EPR systems support the workflow and may ease the burden of documentation and accounting, possibly allowing the GP to spend more time in direct interaction with the patient. However, time studies on physician use of EPRs have failed to demonstrate any noticeable reduction in the time spent on clinician-patient encounters [3-5]. Regarding GPs' attitudes toward EPR systems compared with their paper-based ancestors studies show positive attitudes [6,7], although one study showed clinicians to be far more positive about the quality of paper records than expected [8].

Use of computers may influence the clinician-patient relationship. Some patients may feel reassured by an impression of a greater technical, medical and organizational support given by computers compared to paper folders. On the other hand, the screen may act as a barrier between clinicians and patients. EPRs that do not present reliable or relevant data to clinicians when needed could distract the relationship [9].

In this report we have applied three different methods to study GPs' use of EPR: through focus group interviews, observations of clinical practice, and with use of a questionnaire survey. We have inquired about GPs' use of electronic patient records, measured the actual time spent interacting with the EPRs, and observed and interviewed patients and GPs about the impact of computers on the clinician-patient relationship to find out more about the rapid adoption of Norwegian GP EPR systems.

## Methods

### Setting

Most Norwegian GPs are self-employed and organized in medical practices of an average of 3–4 physicians in a system with enlisted patients. Three different EPR systems offered by two vendors dominate the market (personal communication). Different sections or modules for basic data, medical data, scheduling, financial functions, communications, statistics and other functions build up the EPR systems, but the information is also accessible from a common chronological view of all documentation in the record. The EPR supports most clinical tasks such as free text progress notes, computerized physician order entry, drug prescription, electronic communication, as well as facilitate other functions needed to be independent of paper records. The EPR systems in Norway do not include decision support or instructions on medical procedures.

### Study design

Data was gathered from interviews of GPs in focus groups, from observations of the use of EPR during encounters in clinical practice, and from a questionnaire sent to a random sample of GPs.

### Selection of respondents, data gathering, and analysis

#### Focus groups

Vocational and continuing GP specialist education programs from the Norwegian Medical Association include participation in approved educational groups. We identified some of the groups in the middle of Norway, and invited ourselves to three of them. We chose two continuing groups in the city of Trondheim, and one in the countryside outside Trondheim. The groups represented both GPs with experience with use of paper records and younger physicians with no such experience. There were 23 GPs all together in these groups representing 20 different medical practices. We joined one regular meeting of each of the three groups in 2002 and 2003. The interviews lasted approximately 3 hours. We used an interview guide previously validated by GPs from four different practices and two professors of family medicine. The interviews were recorded on a minidisc with subsequent transcription and later analyzed using NUD*IST Vivo, version 1.1.127. A health secretary familiar with medical terminology transcribed the interviews. Ambiguities were discussed and settled between the secretary, the author (TC) and the co-author (AG). The views expressed across the chosen focus groups were quite consistent and it was considered that more focus groups would not add much additional information.

#### Observations

The observation study was conducted at various periods in 2002, 2003, and 2005. The functions in the EPR systems did not change in this period. A total of 80 GP-patient encounters involving four female and seven male GPs in five medical practices were studied. The practices were strategically chosen to represent all EPR systems. One of the GPs observed had participated in the focus group study. The observed clinicians obtained patient consents prior to each encounter and no patients declined to consent. The observer was situated out of the way behind the patient not to disturb the encounter. Use of different modules or sections in the EPR and time spent on EPR related to some of the encounters were recorded. TC and a sociologist research assistant familiar with observations of health personnel conducted the observations. An observation guide that included a short interview of both patients and clinicians was used after being validated by the researcher, GPs from pilot practices and the supervisor. According to the themes of the study, actual use of EPR was noted with subsequent transcription.

#### Questionnaire

The questionnaire consisted of two major sections and was validated by 20 randomly chosen GPs in a test-retest pilot study in 2002. The respondents in the main study were selected from a database with names and addresses of all GP members of the Norwegian Medical Association and matched with vendor lists of GPs using specific EPR systems. An electronic software program randomly extracted a group of 136 participants from each of the EPR system users. An information letter was sent on February 6th 2003 to all 408 selected GPs, followed by the questionnaire one week later. We collected the last questionnaires in June 2003 after two written reminders followed by three reminders by telephone.

#### Analysis of the collected data

The completed questionnaires were scanned using Teleform and the data were analyzed with SPSS for Windows, version 11.5. Collected material concerning informants' comparative notions of paper records and EPRs, time spent using EPRs during encounters, and effects on clinician-patient relationship was identified and used for systematic text condensation. The analysis of the qualitative material was deductive and the themes and the quotes were derived from the data in four steps: Establishment of a total impression of the material, identification of meaningful units, abstraction of these units, and establishment of the importance of the abstractions [10]. TC coded the transcripts after negotiations with AG and the sociologist researcher, with subsequent definition of the contents of the final categories. The authors' perspective of GPs being responsible for the medical care of enlisted patients supported the analysis. Attention was drawn to the function of EPRs as a tool to support GP medical work, time spent on the EPR, and possible effects on the clinician-patient relationship. Results from the focus groups, observations, and questionnaire survey were compared in the analysis.

## Results

The results from the focus group interviews and the observation study are presented together with relevant data from the questionnaire. Of the 408 GPs invited, 70 were lost due to unknown address, leave of absence, or resignation. Of the 338 GPs who received an invitation, 247 (73%) completed the questionnaire; 18 of the respondents were excluded because they used an older version of the system, used other systems, or EPR system data were missing. Wherever the sample size in the results is other than 229, it is due to missing data. Use of different EPR sections was studied in 53 of the encounters; by this time we were getting results that were very similar to those seen in earlier encounters and we did not consider it necessary to study the use of the different EPR sections in more encounters. Reading in EPR ahead of the encounters was studied in 44 observations. We observed that GPs were using the EPR less than expected from the questionnaire survey, and hence time measurement was added to the last 14 observations. We present the results from all three studies under the same research question headings.

### The availability of individual patient records has improved, but the availability of the information within each record should be better

Saving time looking for patient records, was pointed out by many in the focus groups as a great advantage of EPRs compared to paper records; illustrated by this quote:

The EPR is always available and you can easily maneuver between different records. (No 1)

The focus group interviews revealed that the GPs had almost immediate access to the index pages of different sections in the EPR. However, this access did not imply that access to relevant progress notes and documents was easy. Patient records with many progress notes and documents were often dominated by redundancy of information and the GPs had problems with achieving sufficient overview. Many of the respondents felt it was troublesome to track earlier episodes and notes in the EPR:

My main problem is decreased availability of the information within the EPR in the case of chronically ill patients and patients that have been visiting a number of times. (No 2)

Some of the informants indicated that the overview some times could be better in previous paper records:

"When using paper records we could spread out the papers on the desk to get an overview." (No 3)

Data from the observation study revealed that the GPs rarely spent time searching for historical information in the EPR other than the latest progress notes, medications and results on laboratory tests. Instead, the GPs seemed to rely on their own memory or obtained information through asking the patients about previous episodes. Practically all GPs entered the patient record starting from the list of patients in the appointment book in the EPR. They read the eventual attached remarks or comments made by the health secretary or nurse. GPs read the previous progress note or other parts of the EPR before calling the patient into the office in 36 of 44 observed encounters.

This was partly confirmed by results from the questionnaire study: Practically all respondents (99%) reported to find it useful to check upon previous notes while working with patients; 37% sometimes reported to give up searching for information because it was too time-consuming, and 35% found it easier to ask the patient again rather than to search in the patient record. Almost a third (28%) only occasionally tried to search for information because they found it was too time-consuming. More than half of the respondents (57%) found it difficult to display a summary of the actual progress notes.

### GP use of EPRs seems to be efficient and comprehensive, but also entails administrative tasks previously done by secretaries

The data from the focus groups revealed that a majority of the GPs emphasized the great time and work savings offered by EPR systems compared to paper records. This was exemplified by renewal of regular prescriptions and account keeping, as well as use of text templates and automatic reuse of administrative and clinical information when writing referral letters, requisitions and forms as presented in this quote:

You don't need to write the headings over and over again, and you can also reuse text templates. (No 3)

On the other hand, a shift in administrative workload from health secretaries to GPS was also pointed out in the focus groups. Examples mentioned were scheduling and filling in forms as well as writing referral letters and updating demographic data; illustrated by this quote:

Earlier I dictated referrals. Now I type them myself. (No 4)

**Figure 1:**
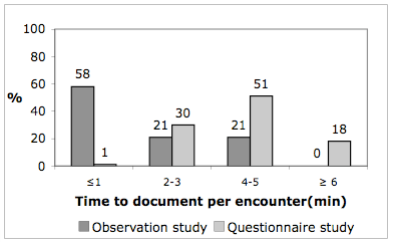
**Time spent to document in EPR**. Differences in time spent to document during an encounter registered in the observation study (No 14) and the questionnaire study (No = 227).

**Figure 2:**
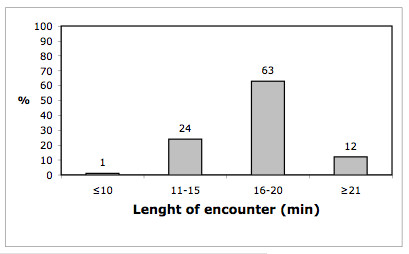
**Length of the encounter**. Distribution of the length of the encounter reported by GPs in the questionnaire study (No = 227).

**Figure 3:**
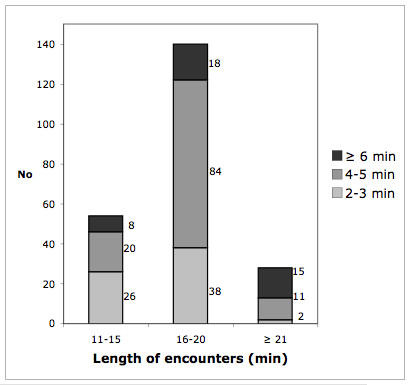
**Length of encounters and time spent to document in the EPR**. Reported by GPs in the questionnaire study (No = 222). One encounter lasting less than 10 minutes and one encounter with documentation time less than one minute were left out from the figure.

These findings were supported by data from the observations. We saw GPs filling in forms, scheduling patients and updating patient contact information, as well as doing all the work surrounding preparation of referral letters. Some even put the referral letter in the envelope themselves (when not sent electronically). We also observed that a few GPs retyped the same information for each referral letter and requisition instead of reusing former information. The use of the EPRs systems was comprehensive. In 53 of the 80 observed encounters, we recorded which EPR sections were in use. We found that 3 to 13 different sections of the EPR were in use during an encounter, with a mean of 6.2 and a median of 6. We also measured the total time spent using the EPR system in 14 of the observed encounters. Data revealed that the time spent registering and documenting in the EPR in the observed encounters was only half of the time compared to what was estimated by respondents in the questionnaire survey (Figure 1). The observed mean time to read an EPR was 49 seconds with a range of 5–150 seconds. According to the questionnaire respondents, encounters lasted between 10 and 21 minutes (Figure 2), and the time recorded in the EPR was related to the encounter time as shown in Figure 3.

### Concerns about the effects of EPRs and computers on the clinician-patient relationship

During the focus group sessions several participants expressed concerns about the potential negative influence of computers on the clinician-patient relationship, particularly when the computer screen drew the GP's attention away from the patient. A majority of the respondents stated that they tried to avoid such disturbances by postponing the documentation in the EPR until after the patient had left. Other GPs claimed to record information during encounters when it seemed to be natural and without disturbance of the relationship. The majority of the GPs claimed that the use of EPRs seldom disturbed the clinician-patient relationship in their opinion, and that working with EPRs was not very different from working with paper records in this respect. Some GPs stated that it was both relevant and useful to conduct documentation work while the patient was still in the room:

When I am not sure if have understood things right; I write the record note while the patient is present, show him the note and ask if it is correctly formulated. (No 5)

In the observation study we interviewed 24 of the patient after the encounters. None of them expressed discomfort with the GP's use of the computer during the encounter nor felt that the screen was an obstacle between them and the clinician. During interviews with all the observed GPs, most of them stated they were aware of the possibility of disturbing their relationship with the patient, and that they tried to avoid such disturbance. We observed that most of the GPs read in the EPR before the encounter began, minimized the use of the EPR during the encounters, and often did the documentation work when the patient had left.

## Discussion

In this study we have found that although the availability of the EPRs was almost immediate, availability of the information within EPRs was not always satisfactory. Use of EPRs was efficient and comprehensive and tightly interwoven with the working processes in their medical practices, but also encompassed more administrative tasks for the physicians compared to paper records. Use of EPRs did not seem to disturb the clinician-patient relationship.

The results indicate that although GP EPR systems are successfully adopted and highly integrated with the clinical work, there are still needs and functionality to be met. The information within the EPR was not always easily available. Instead of looking up information in the EPR, GPs often relied on their own or their patients' memory. This was revealed both in the focus groups, the observations and the questionnaire. Other studies also have confirmed that physicians have greater difficulties in achieving a clinical overview of the situation of the patient when using an EPR system [11].

We found that GPs used the EPR widely and preferred them to paper records. We have in another questionnaire study identified extensive use of EPR with support of 21 of 23 important clinical tasks without need of additional support from paper records. (Paper submitted for publication). Hammond et al. have suggested that clinical information systems do lead to a significant improvement in documentation over handwritten flowsheets, both in volume and accuracy [12]. Other studies suggest that quality improvement is dependent on physicians' use of the EPR system instead of paper for most of their daily tasks [13-15].

We registered that GPs spent less time on reading and recording in the EPR than estimated by the doctors themselves in the questionnaire and that the use of EPR was limited during encounters. Studies support that EPRs can be well-designed and efficient clinical tools [16], but on the other hand EPRs can also become a burden if not well designed [5,17]. This study supports a shift of administrative work from health secretaries to GPs using EPRs compared to paper records. This is in accordance with other studies that have identified greater benefits of EPRs to health secretaries compared to nurses and physicians [18].

In earlier studies patients meant that a computer diminishes the doctor's personal touch and could be regarded as an obstacle to eye contact [19,20]. Our results are in line with more recent studies claiming that well designed EPRs do not disturb the clinician-patient relationship [2].

We believe that the high acceptance and adoption of EPRs in Norway is related to user-centered design, integration, a strong support base of users, and reported improved care quality (personal communication). This is also supported by other studies [21,22]. Other studies report that direct reports and judgments of specific task efficacy from colleagues relate to behavior more often than usability and a general user satisfaction [23]. These factors may also have contributed to the rapid and successful adoption in Norway

In this study both qualitative and quantitative methods were used to obtain data on experiences, behavior and practice processes. We used different methods and in addition observer triangulation to strengthen validity and relevance as well as credibility, confirmability and transferability in the study [24]. The questionnaire gave us representative and sound data on the dissemination of EPR systems and the use of specified clinical tasks, as well as user satisfaction [25]. The interviews uncovered relevant new issues, user experiences and a better understanding of behavior and reactions related to the use of EPRs. We experienced that the observations were preferable to uncover actual use of EPRs during encounters, use of supplementary sources of information, and to study the clinician-patient communication. Although qualitative methods are recommended when evaluating health information systems [26-28], there are several possible limitations to take in account [10]. We believe a combination of methods very often is necessary. Differences in gender or age could possibly introduce biases, but the questionnaire data did not reveal any differences related to gender or age percentiles, and we did not discover any such differences when analyzing the qualitative material either. Although we experienced that common culture and terminology probable eased recruitment of participants and the communication within the focus groups, the authors' previous work as GPs and a background similar to that of the respondents could possibly lead to blind spots or biases when conducting and analyzing the study.

One of the motivations of conducting group interviews was to ensure individual reflections in the groups upon different opinions to ensure internal informant validation. Further validation strategies like negotiations and discussions between TC and AG and research assistants were implemented to avoid errors in the transcription from oral to written information and to validate the findings in both focus groups and observations. Triangulation was carried out in the conduct and analysis of observations to ensure that important or contradictory results related to the research questions were not left out.

The observations revealed issues not thought of when designing a questionnaire. We identified late the need of recording the time used on the EPR during encounters. Time spent on documentation was overestimated by the GPs in questionnaires compared to what we observed. Additional time recordings could have strengthened this discovery. The clinician-patient relationship was another issue not planned for in the questionnaire. Even though the selection of GPs for the focus groups and observations were not randomized, we hold the selection to be representative due to the arbitrary recruitment of different GPs from medical practices in rural and urban districts in the existing groups. We also hold the results to be representative and strengthened when confirmed by several methods.

## Conclusion

Although GPs are generally satisfied with their EPRs systems, there are still unmet needs and functionality to be covered. It is urgent to find methods that can make a better representation of information in large patient records. Further studies are necessary to reveal why and how the introduction of EPRs have increased the administrative workload of physicians and how it could be reduced, as well as clarify contradictory results on time spent on EPR in primary care encounters.

## Competing interests

The author(s) declare that they have no competing interests.

## Authors' contributions

TC planned the investigation and developed the part of the questionnaires used in this study as well as the interview and observation guides with support from his supervisor, AG. TC organized the administration of the questionnaires, focus groups and observations, and analyzed the data with contributions from AG. TC wrote the manuscript with advice from AG.

## Pre-publication history

The pre-publication history for this paper can be accessed here:


